# THE EFFICACY OF CASE-BASED INSTRUCTION ON STUDENT ATTITUDES TOWARDS PAIN AND OPIOID RISK ASSESSMENT IN OLDER ADULTS

**DOI:** 10.1093/geroni/igz038.3211

**Published:** 2019-11-08

**Authors:** Aarushi Aggarwal, Sandra Sanchez-Reilly, Michael J Mader, Jeanette Ross

**Affiliations:** 1 UT Health San Antonio, Long School of Medicine, San Antonio, Texas, United States; 2 South Texas Veterans Health Care System, San Antonio, Texas, United States; 3 South Texas Veterans Health Care System, Dept of Research, San Antonio, Texas, United States; 4 University of Texas Health Science Center San Antonio, San Antonio, Texas, United States

## Abstract

A survey of US medical schools found that only 10-12 hours of the 4 year curriculum are dedicated to instruction in pain management in older adults. Since chronic pain afflicts 100 million Americans, and older adults have a higher risk of prescription medication misuse, there is urgency regarding proper education.This study evaluates the success of case-based instruction on the topics of pain management and opioid risk assessment, with a goal of increasing instructional hours in a format other than didactic. 200 fourth-year medical students were split into groups of 20, with 2 instructors in each room. A survey was administered pre/post workshop asking participants to rate 10 statements using a Likert scale. The 2 hour workshop involved case studies to work through while using a created pain instructional card.The case-based instruction format demonstrated high efficacy in shaping the beliefs and personal evaluations of medical students. Prior to the workshop, only 34% of students were positive about their pain assessment abilities. 9.5% were positive about their opioid conversion skills, and only 4% were positive about opioid risk assessment skills. After the workshop, these positive evaluations increased to 97%, 95%, and 92% respectively. The McNemar test proved these findings to be statistically significant (p<.0001). Case-based instruction with small-group discussion is a reliable tool in teaching medical trainees about pain management and opioid risk assessment in adults aged 65 and older. This workshop needs to be run with geriatric/palliative care residents to evaluate clinical incorporation of session concepts through resident charting.

